# A Systems Approach and Skeletal Myogenesis

**DOI:** 10.1155/2012/759407

**Published:** 2012-09-06

**Authors:** Yoshiaki Ito, Tomohiro Kayama, Hiroshi Asahara

**Affiliations:** ^1^Department of Systems BioMedicine, Graduate School of Medical and Dental Sciences, Tokyo Medical and Dental University, Bunkyo, Tokyo 113-8510, Japan; ^2^Department of Systems BioMedicine, National Research Institute for Child Health and Development, Setagaya, Tokyo 157-8535, Japan; ^3^Department of Orthopaedic Surgery, The Jikei University School of Medicine, Minatoku, Tokyo 105-8461, Japan

## Abstract

Skeletal myogenesis depends on the strict regulation of the expression of various gene subsets. Therefore, the understanding of genome wide gene regulation is imperative for elucidation of skeletal myogenesis. In recent years, systems approach has contributed to the understanding of various biological processes. Our group recently revealed the critical genome network of skeletal myogenesis by using a novel systems approach combined with whole-mount *in situ* hybridization (WISH) database, high-throughput screening, and microarray analysis. In this paper, we introduce our systems approach for understanding the myogenesis regulatory network and describe the advantages of systems approach.

## 1. Introduction

Skeletal muscle is indispensable for any moving function of the human body, and abnormality of the skeletal muscle causes great disability in affected people. It is therefore important to understand the mechanism of skeletal myogenesis so that it may form a basis for disease treatment.

 Almost all skeletal muscles in the body derive from dermomyotome or myotome in somites. The myotome and dermomyotome contain myogenic progenitor cells that evolve into skeletal muscles, aggregates of myofibers, in the whole body. During skeletal myogenesis, myofibers form from myogenic progenitors, where distinct subsets of genes are activated or repressed and form a complex molecular network of interdependent pathways [1–3]. These processes are mainly regulated by the muscle-specific basic helix-loop-helix (bHLH) transcription factors, MyoD, Myf5, Myogenin (Myog), and Mrf4. Analysis of null mice of these genes suggested that MyoD and Myf5 play a role in determining the myogenic progenitors to myoblasts [4]. Myog is important in differentiation from myoblasts to myotubes [5, 6], and Mrf4 is important in both determination and differentiation [7]. The described transcription factors are class II (tissue-specific) bHLH transcription factors capable of either homodimerization or heterodimerization with class I bHLH factors, such as E-proteins HEB/HTF4, E2-2/ITF-2, and E12/E47 [8]. All bHLH dimers bind to an E-box, a consensus sequence comprised of the sequence CANNTG. Id proteins have been identified to act as myogenic antagonists by directly binding to E-proteins and/or muscle-specific bHLH proteins, blocking their ability to bind E-boxes and activate transcription at muscle-specific promoters [9–11]. IdmRNAs are detected in proliferating skeletal muscles and are downregulated in differentiated muscle cultures [9, 12]. This downregulation was thought to be important for skeletal muscle formation; however, the mechanism of Id repression has not been understood for almost 20 years.

 Recently, we revealed the Id downregulation mechanism in myogenesis by our own systems approach combined with WISH database, high-throughput screening, and microarray analysis [13]. Systems approach, a systematic study using various comprehensive analyses such as high-throughput sequencing technologies, genome wide cell-based assays, and bioinformatics, has allowed us to expand our knowledge of life phenomenon. We have reviewed studies that have utilized systems approach. In addition to this, we describe our own systems approach and how this has helped in understanding skeletal myogenesis.

## 2. Deep Sequencing and Array-Based Approaches

High-throughput sequencing technologies allow high-resolution, genome wide investigation of epigenetic conditions. For instance, mapping of open chromatin regions, histone modifications, and DNA methylation across a whole genome is now feasible, and whole transcripts including noncoding RNAs (ncRNAs) can be identified via RNA sequencing.

 These high-throughput sequencing-based technologies and microarray-based ChIP chip analyses are used in various fields, and there have been reports on embryonic stem (ES) cells. Meissner et al. analyzed genome-scale DNA-methylation profiles and histone methylation patterns of mouse ES cells and differentiated cells by using high-throughput bisulphite sequencing and ChIP-sequence [14]. This revealed that DNA methylation patterns are better correlated with histone methylation patterns than with the underlying genome sequence context and that methylation of CpGs is one of dynamic epigenetic marks during differentiation particularly in regulatory regions outside of core promoters [14]. Also, Bock et al. analyzed DNA methylation patterns and gene expression of 20 human ES cell lines and 12 human iPS cell lines, identifying epigenetic and transcriptional similarity of ES and iPS cells [15]. Bernstein et al. mapped Polycomb-associated Histone H3 Lysine 27 trimethylation (H3K27me3) and Trithorax-associated Histone H3 Lysine 4 trimethylation (H3K4me3) across the whole genome in mouse ES cells by ChIP-chip analysis [16]. H3K27me3 is an epigenetic mark that mediates gene silencing, whereas H3K4me3 occurs in nucleosomes found in the promoter regions of actively transcribed genes. They identified a specific modification pattern consisting of large regions of H3K27me3 harboring smaller regions of H3K4me3. It has been proposed that this “active” and “repressive” modification pattern represents genes specifically designed to initiate transcription, and this active state is thought to be essential for the developmental potential of ES cells. Pan et al. also mapped H3K27me3 and H3K4me3 across the whole genome in human ES cells [17]. The vast majority of H3K27me3 colocalized on genes modified with H3K4me3 as within mouse ES cells. These commodified genes displayed low expression levels and were enriched in developmental gene function. Another significant set of genes lacked both modifications, also expressed at low levels in ES cells, but was enriched for gene function in physiological responses rather than development. Commodified genes change expression levels rapidly during differentiation, but so do a substantial number of genes in other modification categories. Pluripotency-associated genes such as SOX2, OCT4, and NANOG shifted from modification by H3K4me3 alone to colocalization of both modifications as they were repressed during differentiation. These data revealed that H3K27me3 modifications change during early differentiation, both relieving existing repressive domains and imparting new ones, and that colocalization with H3K4me3 is not restricted to pluripotent cells. High-throughput sequencing technologies are also used in the studies on genome-wide ncRNA expression analysis. Calabrese et al. analyzed short RNA expression in Dicer-positive and Dicer-knockout mouse ES cells [18]. From quantification of miRNA levels, they estimated that there are 130,000 5′ phosphorylated short RNAs per ES cell. 15% of these RNAs are generated independently of the Dicer gene, presumed breakdown products of mRNAs, which are low in abundance and consist of highly repetitive sequences. The remaining 85% of 5′ phosphorylated ES cell short RNAs consist of miRNAs or miRNA-like species that depend on Dicer for biogenesis. The majority of ES cell miRNAs appear to be generated by six distinct loci, four of which have been implicated in cell cycle control or oncogenesis. At a depth of sequencing that approaches the total number of 5′ phosphorylated short RNAs per cell, miRNAs appeared to be Dicer's only substrate. These studies identified genome-wide epigenetic marks and gene expression in ES cells. They have obtained data revealing the characteristics of ES cells and also incidentally discovered “active” and “repressive” histone comodification patterns. This had been possible due to a genome-wide analysis, thus indicating the importance of such approach.

 The systems approach is also beneficial to reveal the regulatory network of skeletal myogenesis. Myogenesis is orchestrated through a series of transcriptional controls regulated by the myogenic bHLH factors. Several groups performed ChIP-chip analysis to identify targets of myogenic regulatory factors [1, 2]. These analyses indicated overlapping of distinct targets of MyoD and Myog suggesting the mechanism of sequential expression during myogenesis. At early myogenesis, MyoD is sufficient for activation of the expression, and these genes are expressed immediately after MyoD induction. On the other hand, during late myogenesis, MyoD initiates only regional histone modification. Myog does not bind without MyoD, and the expression of late genes requires both MyoD and Myog. In recent years, genome-wide MyoD target profiling using ChIP-sequence analysis has been reported [19]. High-throughput sequencing technology-based ChIP-sequence analysis suggested over 20,000 MyoD-binding sites, greater than with the array-based ChIP-chip analysis [19]. This analysis identified that MyoD was constitutively bound to thousands of sites in both myoblasts and myotubes and that the genome wide MyoD binding was related with regional histone acetylation [19]. This suggests that myogenic master regulator MyoD genome widely acts to alter the epigenome in myoblasts and myotubes. Gagan et al. also performed high-throughput sequencing-based analysis, to find that MyoD binds to the microRNA-378 (miR-378) gene locus and induces transactivation and chromatin remodeling [20]. This activated miRNA directly downregulates the MyoR, a MyoD antagonist, and promotes myogenesis [20].

 Genome-wide target gene analyses are also performed in transcription regulators other than the myogenic bHLH factors. Lagha et al. performed ChIP-chip analysis of the transcription factor Pax3 [21], which is essential for ensuring myogenic potential and survival of the progenitors [22]. Pax3 binds to a sequence 3′ of the Fgfr4 gene that directs Pax3-dependent expression at sites of myogenesis in transgenic mouse embryos. The activity of this regulatory element is also partially dependent on E-boxes, targets of the myogenic regulatory factors, which are expressed as progenitor cells entering the myogenic program. Other FGF signaling components, notably Sprouty1, are also regulated by Pax3. These results provide new insight into the Pax-initiated regulatory network that modulates stem cell maintenance versus tissue differentiation. Soleimani et al. performed ChIP-seq analysis of Pax3 and Pax7 [23]. These transcription factors regulate stem cell function in skeletal myogenesis, but little is known about the molecular mechanism of their distinct roles. The genome-wide binding-site analysis combined with gene expression data indicates that both Pax3 and Pax7 bind identical DNA motifs and jointly activate a large number of genes involved in muscle stem cell function. In adult myoblasts, Pax7 binds to many more sites than the number of genes it regulates. In spite of a significant overlap in their transcriptional network, Pax7 regulates distinct set of genes involved in the acceleration of proliferation and inhibition of myogenic differentiation. Moreover, they showed that Pax7 has a higher binding affinity to the homeodomain-binding motif relative to Pax3, suggesting that the differences in DNA binding contribute to the observed functional difference between Pax3 and Pax7 binding in myogenesis. Mousavi et al. performed ChIP-seq of Polycomb group (PcG) protein Ezh1 and mRNA-seq in skeletal muscle cells [24]. This study provides evidence for genome-wide association of Ezh1 complex with active epigenetic mark (H3K4me3), RNA polymerase II (Pol II), and mRNA production. Although Ezh2, a paralog of Ezh1, is a known trigger for transcription repression by catalyzing the addition of methyl groups onto H3K27 [25], these findings reveal another role for PcG complex in promoting mRNA transcription.

 The genome-wide approach also contributes to further understanding of the epigenetic regulation in skeletal myogenesis. Asp et al. examined changes in the chromatin landscape during myogenesis by ChIP-seq analyses of several key histone marks (H3K9Ac, H3K18Ac, H4K12Ac, H2Bub, H3K4me1, H3K4me2, H3K4me3, H3K27me3, and H3K36me3) and RNA polymerase II in mouse myoblasts and myotubes [26]. Using the data, they identified novel regulatory elements flanking the *Myog* gene that act as a key differentiation-dependent switch in myogenesis. *Myog* gene is targeted by PRC2-mediated H3K27 methylation, and its expression is suppressed in myoblasts. Depletion of Suz12, a component of PRC2 complex that regulates H3K27 methylation, led to the loss of PRC2 and H3K27me3 on Myog, resulting in premature and enhanced gene induction. This histone mark could represent part of a methylation-acetylation differentiation switch, determining the timing of expression of *Myog* and therefore terminal differentiation. Vethantham et al. also performed ChIP-seq analyses of H2Bub, H3K4me3, and H3K79me3 during myogenesis [27]. Ubiquitylation of H2B on lysine 120 (H2Bub) is associated with active transcriptional elongation. H2Bub has been implicated in histone crosstalk and is generally thought to be a prerequisite for trimethylation of H3K4 and H3K79 in both yeast and mammalian cells. The genome-wide analysis of epigenetic marks identified dynamic loss of H2Bub in the differentiated state. Moreover, they found that the H2B ubiquitin E3 ligase, RNF20, was depleted from chromatin in differentiated myotubes, indicating that recruitment of this protein to genes significantly decreases during myogenesis. Furthermore, they observed retention and gaining of H3K4 trimethylation on multiple genes in the absence of H2Bub. The Set1 H3K4 trimethylase complex was efficiently recruited to a subset of genes in myotubes in the absence of H2Bub, suggesting that H3K4me3 in the absence of H2Bub in myotubes is mediated via Set1.

 Trapnell et al. performed RNA-seq analysis in mouse myoblast cell line representing a differentiation time series [28]. They detected 13,692 known transcripts and 3,724 previously unannotated transcripts. Analysis of transcript expression over the time series revealed complete switches in the dominant transcription start site or splice isoform in 330 genes, along with more subtle changes in further 1,304 genes.

 Overall, deep sequencing or array-based approaches have been shown to be of benefit in identifying the molecular network and novel effectors in diverse biological processes. In skeletal myogenesis, these approaches revealed comprehensive target genes of myogenic transcription factors, novel myogenic factors and the characteristics of myoblasts and myotubes, which could not be identified by conventional approaches.

## 3. Cell-Based High-Throughput Assay

Currently, multiple studies have demonstrated comprehensive and cell-based functional screening. Generally, screening for signals activating gene expression consists of examining potential transcription factor-binding sequences in a specific promoter using bioinformatics and reporter assays. If a factor's potential recognition motif is unknown, one-hybrid or South-western screening can be used to identify molecules directly associated with the specific sequence. However, these methods are limited to identifying direct targets only. On the other hand, cell-based reporter assays using a comprehensive set of cDNAs in an expression library allow high-throughput screening not only for direct transcriptional regulators but also for other factors, such as cell-signaling molecules, receptors, and growth factors. Chanda et al. performed a reporter assay-based approach that used about 20,000 annotated cDNAs in the investigation of activator protein-1 (AP-1) signal transduction pathway and identified novel factors of AP-1 mediated growth and mitogenic response pathway [29]. Fiscella et al. performed high-throughput assay using a unique library of cDNAs encoding predicted secreted and transmembrane domain-containing proteins [30]. Supernatants from mammalian cells transiently transfected with this library were incubated with primary T cells and T cell lines in several high-throughput assays including reporter and cytokine secretion assay. This identified a T cell factor, TIP (T cell immunomodulatory protein), which does not show any homology to proteins with known function. However, treatment of primary human and murine T cells with TIP resulted in the secretion of IFN-*γ*, TNF-*α*, and IL-10, whereas in vivo TIP had a protective effect in a mouse acute graft-versus-host disease (GVHD) model. Konig et al. performed a systematic approach combined with genome-wide siRNA analysis and searched the human interactome database, to uncover multiprotein virus-host interactions that are likely to regulate the early steps of HIV infections [31].

 In the myogenesis study, we performed cell-based high-throughput transfection assay to identify activation factors of RP58, a critical myogenesis regulator as described in the latter section.

## 4. *In Situ* Gene Expression Database

Microarray analysis is a powerful tool to identify the working genes in individual cells or tissues. However, this analysis is unlikely to detect gene expression restricted to small areas. In contrast, *in situ* hybridization can identify temporal and spatial gene expression patterns. The systematic *in situ* hybridization database contributes to detailed information for the spatial regulation of gene expression. Gray et al. mapped the expression of 1174 transcription factors in the brain of developing mice using section *in situ* hybridization [32]. Also, Lein et al. described an anatomically comprehensive digital atlas containing the expression patterns of around 20,000 genes using automated high-throughput procedures for *in situ* hybridization in the adult mouse brain [33]. These databases describe the anatomical organization of the brain and provide a primary data resource for a wide variety of further studies regarding brain organization and its function.

 The Edinburgh Mouse Atlas Gene-Expression Database (EMAGE) is a large-scale database of *in situ* gene expression patterns of about 16,000 genes in the developing mouse embryo [34–37]. Domains of expression from raw data images are spatially transferred into a set of standard 3D virtual mouse embryos at different stages of development. Anatomy ontology is also used to describe sites of expression, which allows data to be queried using text-based methods. The GenitoUrinary Development Molecular Anatomy Project (GUDMAP) is also a database of *in situ* gene expression patterns in mouse embryos [38, 39]. GUDMAP includes whole-mount and section *in situ* hybridization data of over 3,000 genes and microarray gene expression data of microdissected, laser-captured, and FACS-sorted components of the developing mouse genitourinary (GU) system. These *in situ* gene expression databases provide more detailed information on the spatial regulation of gene expression and allow identification of discrete clusters of transcribed genes. They serve as a useful source for research in developmental biology.

## 5. Our Systems Approach Revealed the MyoD-Mediated Ids Repression Mechanism

We constructed a unique systems approach and applied it for elucidation of myogenesis molecular network. First, we created our own *in situ* gene expression database. To identify and characterize effectors of the transcriptional network regulating developmental processes, we developed a web-based comprehensive WISH database for transcriptional regulators using E9.5, 10.5, and 11.5 mouse embryos [13]. We prepared 1520 digoxigenin-labeled RNA probes from cDNA libraries. Using WISH results, we annotated gene expression patterns of each gene and constructed a database, termed “EMBRYS” (http://embrys.jp/embrys/html/MainMenu.html), covering these 3 embryonic days. Using this database, we identified 43 transcription regulators showing myogenic expression pattern in the limb bud. Among those, transcription repressor *RP58* was identified as a novel transcription factor expressed in myogenesis [13]. The analysis of *RP58* knockout mice revealed that this gene is critical for myogenesis [13]. This database EMBRYS is also useful to identify regulators of another tissue development. Indeed, we also identified that *Mohawk homeobox* gene is a critical regulator of tendon differentiation by using the database [40].

 The WISH database EMBRYS identified a novel transcriptional factor RP58 as a critical regulator of myogenesis. To identify the molecular network anchored by RP58, we investigated the upstream events that promote RP58 expression by cell-based expression vector library transfection assay [13]. We utilized around 6000 arrayed and addressable cDNA clones, which allowed systematic, efficient, and unbiased screening of cDNA encoding factors that could activate the RP58 promoter. A highly conserved RP58 genomic region was inserted in front of luciferase gene in the reporter vector. This was then transfected in 293T cells with expression vector library, and luciferase assay was performed [13]. The high-throughput transfection assay identified myogenic bHLH factor MyoD as a direct transcription activator of RP58 [13].

 RP58 has been reported to bind to the specific DNA sequence (A/C)ACATCTG(G/T)(A/C) [41] and is associated with Dnmt3a and Hdac1 [42]. These reports suggest that RP58 can bind to the promoter region of its target genes and repress transcription activity. To identify the repression targets of RP58, we performed microarray analysis and bioinformatics screening by RP58 binding sequence and identified Id2 and Id3 as RP58 repression targets [13].

**Figure 1:**
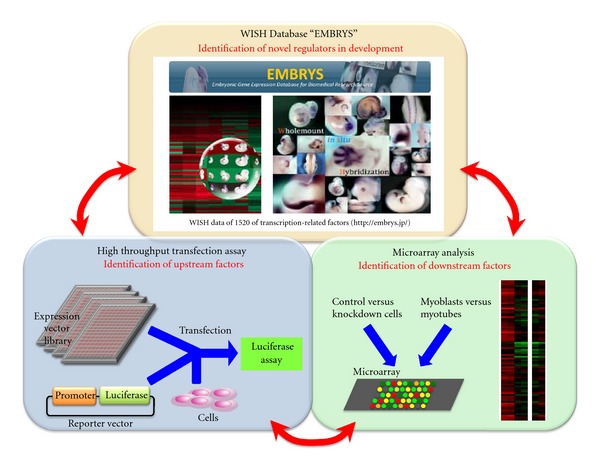
Scheme of our systems approach. WISH database EMBRYS identified a novel myogenesis regulator RP58. High-throughput transfection assay and microarray analysis identified upstream and downstream factors of RP58. This multicombined approach is useful for elucidation of molecular network in the developmental process.

**Figure 2:**
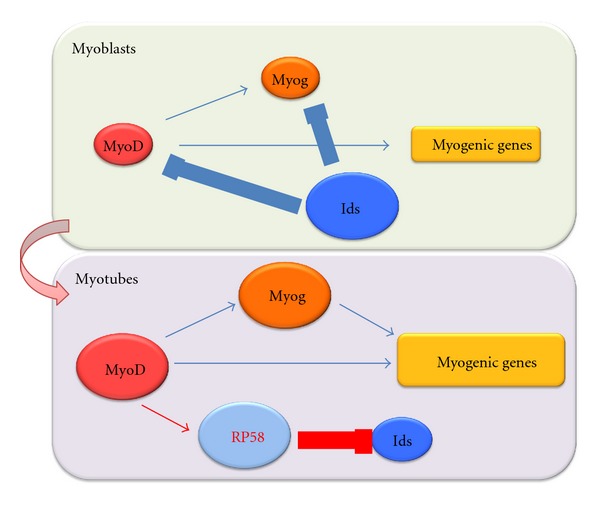
Proposed myogenesis regulatory network by our systems approach in myoblasts; Id proteins are expressed and inhibit the myogenic bHLH factors. During myogenic differentiation, RP58 is promoted by MyoD and represses the Id transcription. Muscle specific genes are then activated by myogenic bHLH factors.

 Our systems approach combined with WISH database construction, high-throughput transfection assay, and microarray analysis identified a critical regulatory network of myogenesis (Figure 1). WISH database identified a novel myogenic regulator, RP58. High-throughput transfection screening and microarray analysis identified a MyoD-activated regulatory loop by RP58-mediated repression of myogenic bHLH factor inhibitors Id2 and Id3. In myoblasts, Ids are expressed and inhibit the myogenic bHLH factors. During myogenesis, RP58 is promoted by MyoD and represses Id transcription. Myogenesis then progresses by myogenic bHLH factor-mediated activation of muscle-specific genes (Figure 2). The repression mechanism of Ids had been unclear for almost 20 years, and this new finding indicates the importance of this systems approach.

## 6. Conclusion

A genome-wide systematic approach using high-throughput sequencing technologies, cell-based transfection assays, or construction of gene expression pattern database is contributing to understanding the mechanisms of various life phenomena. These methods have also been shown to be useful in studying skeletal myogenesis. High-throughput sequencing-based technologies showed genome-wide target genes of myogenesis regulators and epigenetic modification in skeletal myogenesis. We also identified a novel myogenesis network regulated by RP58 using the multicombined approach. Although the myogenesis study using systems approach is still at its early stages, the systems approach will enable further understanding of myogenesis in the future.
